# Predicting trajectories of recovery in prostate cancer patients undergone Robot-Assisted Radical Prostatectomy (RARP)

**DOI:** 10.1371/journal.pone.0214682

**Published:** 2019-04-04

**Authors:** Chiara Marzorati, Dario Monzani, Ketti Mazzocco, Francesca Pavan, Gabriele Cozzi, Ottavio De Cobelli, Massimo Monturano, Gabriella Pravettoni

**Affiliations:** 1 Department of Oncology and Hemato-Oncology, University of Milan, Milan, Italy; 2 Applied Research Division for Cognitive and Psychological Science, European Institute of Oncology IRCCS, Milan, Italy; 3 Patient Safety & Risk Management Service, European Institute of Oncology IRCCS, Milan, Italy; 4 Division of Urology, European Institute of Oncology IRCCS, Milan, Italy; University of South Alabama Mitchell Cancer Institute, UNITED STATES

## Abstract

**Objective:**

To identify trends of patients’ urinary and sexual dysfunctions from a clinical and psychological perspective and understand whether sociodemographic and medical predictors could differentiate among patients following different one-year longitudinal trajectories.

**Methods:**

An Italian sample of 478 prostate cancer patients undergone Robot-Assisted Radical Prostatectomy completed the EPIC-26 survey between July 2015 and July 2016 at the pre-hospitalization (T0), 45 days (T1) and 3 (T2), 6 (T3), 9 (T4), and 12 months (T5) after surgery. Sociodemographic and clinical characteristics (age, BMI, diabetes, nerve-sparing procedure) were also collected. Latent Class Growth Analysis was conducted separately for sexual dysfunction and urinary incontinence EPIC-26 subscales. The association between membership in the two longitudinal trajectories of urinary and sexual dysfunctions was assessed by considering Chi-square test and its related contingency table.

**Results:**

People who have a high level of urinary incontinence at T1 are likely to have a worse recovery. Age, BMI and pre-surgical continence may affect the level of incontinence at T1 and the recovery trajectories. Patients with low and moderate sexual problems at T1 can face a moderate linear recovery, while people with high level of impotence immediately after surgery may take a longer period to solve sexual dysfunctions. Age and the pre-surgical sexual condition may impact the recovery. Finally, a great proportion of patients reported both steady problems in sexual function and constant high levels of urinary incontinence over time.

**Conclusions:**

This study highlights different categories of patients at risk who may be important to know in order to develop personalized medical pathways and predictive models in a value-based healthcare.

## Introduction

Prostate cancer (PC) is diagnosed in over one million of people worldwide and almost 70% of these cases occurred in the developed regions, where tumor detection and patients’ life span are prolonged by advancement in treatments [1–3]. In fact, the 5-year survival was estimated roughly 80–90% in most of the European countries, except those in Eastern Europe [4]. Therefore, cancer screening and new interventions have become increasingly wide in order to successfully treat the disease, with less side-effects and improving patients’ quality of life. According to the specific diagnosis, localized PCs are treated with radiotherapy, active surveillance, and, above all, radical prostatectomy[5–7]. Despite all advancements in surgical and radiant treatments, a recent study shows that, regardless any treatments, 90% of PC survivors reported at least one physical impairment during the two-years post-diagnosis. Sexual, urinary and bowel dysfunctions are the most common functional consequences after PC treatments and they may persist for several years [8,9]. Compared to radiotherapy and active surveillance, men who underwent robot-assisted radical prostatectomy (RARP) reported greater negative side effects especially in the first 6 months from the surgery, even though there was a partial recovery [10]. Sixty percent of men suffer from urinary incontinence and sexual problems after surgery, that are often related to distress and fatigue symptoms. Following a variable-oriented approach, several studies investigate the trend of these symptoms showing that patients live longer with these side effects that deeply affect their quality of life for months after treatment [11–15]. Nevertheless, it would be also important to identify different categories of PC patients with different trends in recovery over time through a person-oriented approach that can better display which kind of patients may have higher difficulties in the recovery of their functional and psychological abilities. The person-oriented approach is a valid alternative to the traditional variable-oriented methodology. The main analytic units of the variable-oriented approach in health research are individual behaviours, health-related variables or construct that may vary both within populations or over time [16]. In this kind of approach inter-individual differences are rarely taken into account because they are considered random and negligible [17]. On the contrary, the main assumption of the person-oriented approach is that people are unique and that this uniqueness is measurable and worth knowing [18]. The main analytic units of this approach are individuals or homogeneous subpopulations. From this perspective, inter-individual differences are meaningful and may be especially helpful to classify individuals into distinct classes. Individuals belonging to the same class are similar to each other while they are quite different from those classified in other classes. Examples of the recent application of the person-oriented approach in health research are the identification of: specific disease-prone personality types [19–21], longitudinal trajectories of neuropsychological symptoms and cognitive decline in patients with Alzheimer’s diseases [22,23], longitudinal pattern of change of lifestyle following acute coronary syndrome [24,25], and determinants of trajectories of quality of life in people with type 2 diabetes [26]. Concerning prostate cancer, Chambers et al. (2017) [27] applied a person-oriented approach to understand which factors can affect different trajectories of patients’ recovery in Quality of Life, life satisfaction and psychological adjustment after different prostate cancer treatments, but nobody has already focused the study research on physical function’s recovery in men with prostate cancer after RARP surgery.

Clinical and sociodemographic characteristics may foreshadow the partial recovery of men treated with RARP. Different studies showed that age, body mass index (BMI), and diabetes may predict long-term post-operative incontinence after RARP [6,28]. While preservation of the neurovascular bundle, age, and pre-surgery sexual condition were considered as positive predictors of potency recovery following RARP [29–31]. Therefore, patients’ characteristics play an important role in months after surgery: some pre-intervention sociodemographic and clinical variables may influence and predict typical trend of patient’s recovery after robot-assisted radical prostatectomy.

Physical dysfunctions are normally present during the months after surgery, highly affecting patient’s survivorship and Quality of Life [12]. This new study perspective may help people involved in the care process to better identify possible trajectories of physical and psychological outcomes and predict which of the identified categories of patients would have greater difficulties in their recovery.

The aim of this research study is to identify one-year trends of patients’ urinary and sexual dysfunctions from a clinical and psychological point of view and understand whether sociodemographic (i.e., age) and medical variables (i.e., diabetes, BMI, nerve sparing, pre-surgery scores of urinary incontinence and sexual dysfunction) could differentiate among patients following these different one-year longitudinal trajectories.

## Materials and methods

### Participants and procedure

An Italian sample of 478 men with localized PC who participated in the Value Based Project and undergone RARP were enrolled at the European Institute of Oncology in Milan between July 2015 and July 2016. Patients were included in the study if they: 1) were diagnosed with localized PC, 2) were native Italian speakers, 3) referred to the Value Based Project and 4) had not neurological or psychopathological problems. All eligible men were firstly asked to give written informed consent and then were asked to complete the self-report EPIC-26 survey. At the pre-intervention, sociodemographic and clinical characteristics were also collected, in particular age, BMI, presence or not of diabetes, PSA Class, Charlson Comorbidity Index, Gleason Score, ASA Class, and preservation or not of the neurovascular bundle (nerve-sparing procedure). Clinical characteristics were described in Table 1. They completed the EPIC-26 questionnaire at the pre-hospitalization (T0), 45 days (T1) and 3 (T2), 6 (T3), 9 (T4), and 12 months (T5) after RARP surgery. Since our aim was to study the trend of sexual and urinary dysfunction after surgery and we saw that there was low outcomes variability among patients before surgery, we excluded the baseline outcomes and run the analyses starting from T1, that is 45 days after prostatectomy. Baseline outcomes (i.e., urinary incontinence and sexual dysfunction), alongside with sociodemographic and other clinical variables, were used to predict patients’ membership in the identified longitudinal trajectories of urinary incontinence and sexual dysfunction over time. All information and data were collected and analyzed by a multidisciplinary team of the Value Based Project. The Ethical Committee of the European Institute of Oncology approved the study.

**Table 1:** Sample clinical characteristics.

	Sample (%)
***Pre-surgery variables***	
**Gleason Score**	
≤ 6	46.1
7	39.4
8	10.7
9–10	3.8
**PSA Class**	
Less than 4	12.8
4–10	66.9
More than 10	20.3
**ASA Class**	
1	18.7
2–3	81.3
**BMI Class**	
Normal (< 27)	66.7
Overweight (≥27)	33.3
**Charlson Index**	
<1	72.8
≥1	27.2
***Surgery variables***	
**Nerve Sparing**	
No	26.4
Unilateral	55.9
Bilateral	17.7
***Post-surgery variables***	
**Complications**	
No	89.3
Yes	10.7

### Measures

The Expanded Prostate Cancer Index Composite—Short Form EPIC-26 is the most used cancer-specific survey in Urology divisions to measure patient’s well-being [32–34]. The EPIC-26 is a brief self-report scale, collecting medical and psychological information on urinary incontinence, urinary irritation, bowel, sexual and hormonal dysfunction with a Likert-scale from 0 to 4 (or 5 in some items). Urinary Incontinence subscale consists of 4 items investigating leaking urine, urinary control, number of pads used per day and overall urinary functioning, in the last 4 weeks. Sexual dysfunction includes items on the ability to have an erection, ability to reach an orgasm, quality of erections, frequency of erections, and overall sexual function, in the last 4 weeks. Higher scores in subscales indicate worst medical conditions or higher problem perception. The EPIC-26 was administered at the pre-hospitalization (T0), 45 days (T1) and 3 (T2), 6 (T3), 9 (T4), and 12 months (T5) after RARP surgery.

Age and BMI were collected for each participant at the pre-hospitalization. According to the WHO Guidelines [35], a BMI cut-off of 27 divided the sample into two classes: patients with a BMI< 27 were included in the “normal weight” class, while those with a BMI≥27 were included in the “overweight” class.

Diabetes and the preservation of the neurovascular bundle were included in medical variables. Three classes were identified: 1) patients undergone to radical prostatectomy with bilateral nerve sparing procedure, 2) patients undergone to radical prostatectomy with unilateral nerve sparing, and 3) patients undergone to radical prostatectomy with no nerve sparing.

### Statistical analysis

To identify different longitudinal trajectories of patients with PC undergone RARP based on their initial status and change over time in urinary incontinence and sexual dysfunction, we performed a Latent Class Growth Analysis (LCGA) conducted separately for each of these two EPIC-26 subscales. The LCGA is a flexible methodology to model patient longitudinal trajectories from unobserved subpopulations (i.e., latent trajectory classes) with patient variation in growth parameters (e.g., intercept and slope) that are expressed with random effects. Another advantage of this methodological approach is that predictors of longitudinal trajectory membership could be identified within the LCGA framework by directly introducing these independent variables in the model. This permitted to quantify the net effect of each predictor whist adjusting for the other ones and, thus, to better and more validly identify the best predictors of longitudinal trajectory membership (for a brief and clear overview of LCGA, see Jung & Wickrama, 2008)[36]. The LCGA approach has been efficiently adopted to identify trajectories of change over time in quality of life, symptomatology, and adjustment to several types of illness, such as heart failure [37], unipolar depression [38], low back pain [39], and breast cancer [40].

Non-linear LCGA consisting of intercept, slope and quadratic growth parameters were performed with Mplus 8.2. Missing urinary incontinence and sexual dysfunction across waves were handled using a robust full information maximum likelihood (FIML) estimation procedure. To determine the number of classes to be extracted, we primarily considered the Lo-Mendell-Rubin likelihood ratio test (LMR-LRT) following by other statistical considerations, such as a successful convergence (i.e., no local maximum likelihood), high entropy value close to 1 (i.e., high precision and certainty in the classification), and total count within each classes above 1% (i.e., absence of classes with too few members). Specifically, the LMR-LRT compares the solution with *k* classes with the solution with *k*-1 classes; statistically significant values indicate that the broader solution (i.e., *k* classes) better fits the data than the more restricted *k*-1 classes solution. After determining the number of classes to be extracted for the urinary incontinence and sexual dysfunction, clinical and sociodemographic predictors of longitudinal change membership were introduced in the unconditional LCGA models via multinomial logistic regression. We compared the reference class (i.e., high levels of urinary incontinence or sexual dysfunction) with the other identified longitudinal trajectories to assess the discriminative power of each clinical and sociodemographic predictor in differentiating among the identified longitudinal trajectories. Specifically, pre-surgery urinary incontinence score, age, BMI (0 = BMI less than 27; 1 = BMI equal or greater than 27), diabetes (0 = no diagnosis of diabetes; 1 = diagnosis of diabetes) were introduced as predictors to explain membership in longitudinal trajectories of urinary incontinence. Pre-surgery sexual dysfunction score, age, and nerve sparing were introduced as predictors to explain membership in longitudinal trajectories of sexual dysfunction. Because Mplus does not accommodate categorical independent variables, nerve sparing was entered as two distinct dummy variables (i.e., unilateral and bilateral nerve sparing; no nerve sparing was the reference category). In each model, the worst longitudinal trajectory was chosen as the reference category in the multinomial regression model.

Finally, the association between membership in the two longitudinal trajectories of urinary incontinence and sexual dysfunction was assessed by considering results of a Chi-square test and its related contingency table.

## Results

### Identification of longitudinal trajectories of urinary incontinence

**Fig 1:**
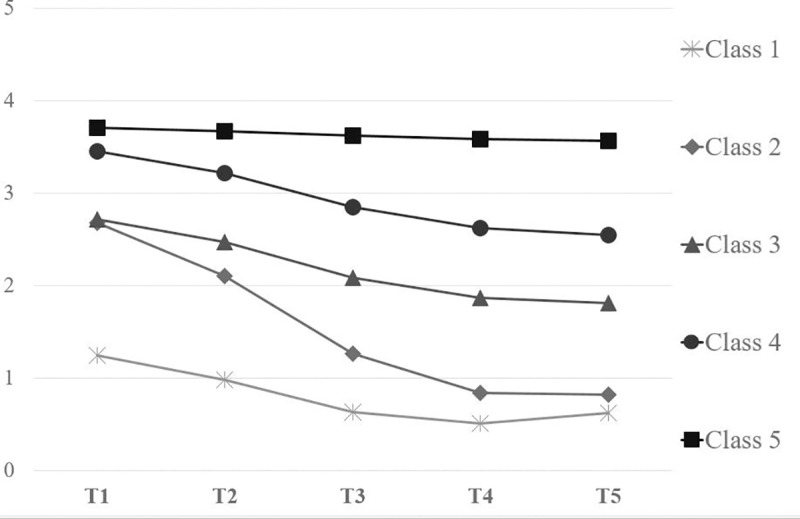
The five identified longitudinal trajectories of urinary incontinence.

LCGA was performed on the urinary incontinence scores of the EPIC-26 measured at the five time points of the present study. A five-class model with five different longitudinal trajectories was chosen because the LMR-LRT indicated that the five classes are significantly better than four (p = .039) and better than six (p = .266). Moreover, this five-class solution also displayed a high entropy level (.837) and total count within each class above 1% (min = 4.65%; max = 38.44%). Fig 1 reports the five identified longitudinal trajectories of urinary incontinence over time.

Patients in the first class (38.44% of the total count) showed the lowest initial status of urinary incontinence (intercept = 1.25, p < .001) and a moderate recovery over time (slope = -0.29, p < .001; quadratic = 0.03, p < .001). We labeled this longitudinal trajectory as “Class 1”. As shown in Table 2, 51.4% of patients presented with biopsy Gleason score 6, while in 2.2% of cases was higher than 8. The majority of patients (67%) had a pre-surgical PSA between 4 and 10, and 76.1% had an ASA score between 2 and 3. The Charlson Index was lower than 1 in 78.4% of the sample. Moreover, 91.4% of patients who underwent RARP and belonging to this Class had not post-surgical complications. The “Class 2” encompassed 26.44% of patients with moderate initial levels of urinary incontinence (intercept = 2.68, p < .001) and a sudden decrease of symptomatology over time (slope = - 0.62, p < .001; quadratic = 0.05, p < .001). Eighty-five percent of patients belonging to Class 2 had a Gleason Score lower than eight, 73.1% of them had a PSA index varying between 4 and 10, and 84.2% an ASA score of 2 or 3. Their Charlson Index was lower than 1 in 72.4% of the cases and 89.6% of the Class 2 had not complications after surgery (see Table 2). The third class (18.32% of the total count), labeled “Class 3”, was characterized by a moderate urinary incontinence at baseline (intercept = 2.72, p < .001) and a moderate recovery over time (slope = - 0.27, p < .001; quadratic = 0.02, p = .021). Almost 49% of patients presented with biopsy Gleason score 6, while in 1.2% of cases was 9 or 10 (see Table 2). The majority of patients (60%) had a pre-surgical PSA between 4 and 10, and 83.5% had an ASA score between 2 and 3. The Charlson Index was lower than 1 in 68.8% of the sample. Moreover, 88.8% of patients who underwent RARP and belonging to Class 3 had not post-surgical complications. The “Class 4” included 12.14% of patients with a high urinary incontinence at 45 after the RARP (intercept = 3.46, p < .001) and a moderate recovery over time (slope = - 0.26, p < .001; quadratic = 0.02, p = .059). Eighty-three percent of patients belonging to Class 4 had a Gleason Score lower than eight, 59.3% of them had a PSA index varying between 4 and 10, and 84.5% an ASA score of 2 or 3. Their Charlson Index was lower than 1 in 71.2% of the cases and 88.1% of the Class 4 had not complications after surgery (see Table 2). The last class (4.65% of the total count) had the worst initial status of urinary incontinence (intercept = 3.70, p < .001) with a flat and non-significant linear and quadratic trajectory (linear slope = - 0.03, p = .403; quadratic slope = 0.00, p = .758). This class could be labeled “Class 5”. As shown in Table 2, this Class showed a Gleason Score of 6 in 35% and 7 in 40% of the cases, respectively. Seventy-five percent of the patients belonging to Class 5 had a PSA index varying from 4 to 10, and 90% of them showed an ASA score of 2 or 3. The Charlson index was higher than 1 in 55% of the cases and men of this Class had post-surgical problems in 25% of the cases.

**Table 2:** Clinical variables in identified urinary clusters.

Clinical Variables	Class 1 (%)	Class 2 (%)	Class 3 (%)	Class 4 (%)	Class 5 (%)
**Gleason Score**					
≤ 6	51.4	42.5	48.8	39.0	35.0
7	35.5	42.5	40.0	44.0	40.0
8	10.9	7.5	10.0	13.6	25.0
9–10	2.2	7.5	1.2	3.4	0
**PSA Class**					
Less than 4	14.6	7.5	18.8	11.9	10.0
4–10	67.0	73.1	60.0	59.3	75.0
More than 10	18.4	19.4	21.2	28.8	15.0
**ASA Class**					
1	23.9	15.8	16.5	15.5	10.0
2–3	76.1	84.2	83.5	84.5	90.0
**Charlson Index**					
<1	78.4	72.4	68.8	71.2	45.0
≥1	21.6	27.6	31.2	28.8	55.0
**Complications**					
No	91.4	89.6	88.8	88.1	75.0
Yes	8.6	10.4	11.2	11.9	25.0

### Sociodemographic and clinical predictors of longitudinal trajectories of urinary incontinence

Then, the predictive role of clinical and sociodemographic variables to explain membership in longitudinal trajectories of urinary incontinence was assessed through multinomial logistic regression. Specifically, pre-surgery urinary incontinence score, age, BMI, and diabetes were introduced in this model as predictors of longitudinal change membership.

Because “Class 5” was the worst longitudinal trajectory of urinary incontinence over time, this class was chosen as the reference category in the multinomial regression model. Results showed that more elderly patients (B = -0.09, OR = 0.92, p = .004) and higher levels of pre-surgery incontinence (B = - 1.30, OR = 0.27, p = .003) had a lower chance to belong to “Class 2” compared to “Class 5”. Moreover, compared to “Class 5”, “Class 4” was characterized by overweight or obese patients (B = 1.04, OR = 2.83, p = .035). More elderly patients (B = - 0.08, OR = 0.92, p = .007) with higher levels of pre-surgery incontinence (B = - 2.27, OR = 0.10, p = .015) had a lower likelihood of being included in “Class 1” than in “Class 5”. Finally, compared to the worst longitudinal trajectory of urinary incontinence over time, prostate patients within “Class 3” were younger (B = - 0.08, OR = 0.92, p = .025) and with less pre-RARP incontinence (B = - 1.11, OR = 0.33, p = .007).

Diabetes was not helpful to distinguish between patients belonging to the five classes.

### Identification of longitudinal trajectories of sexual dysfunction

**Fig 2:**
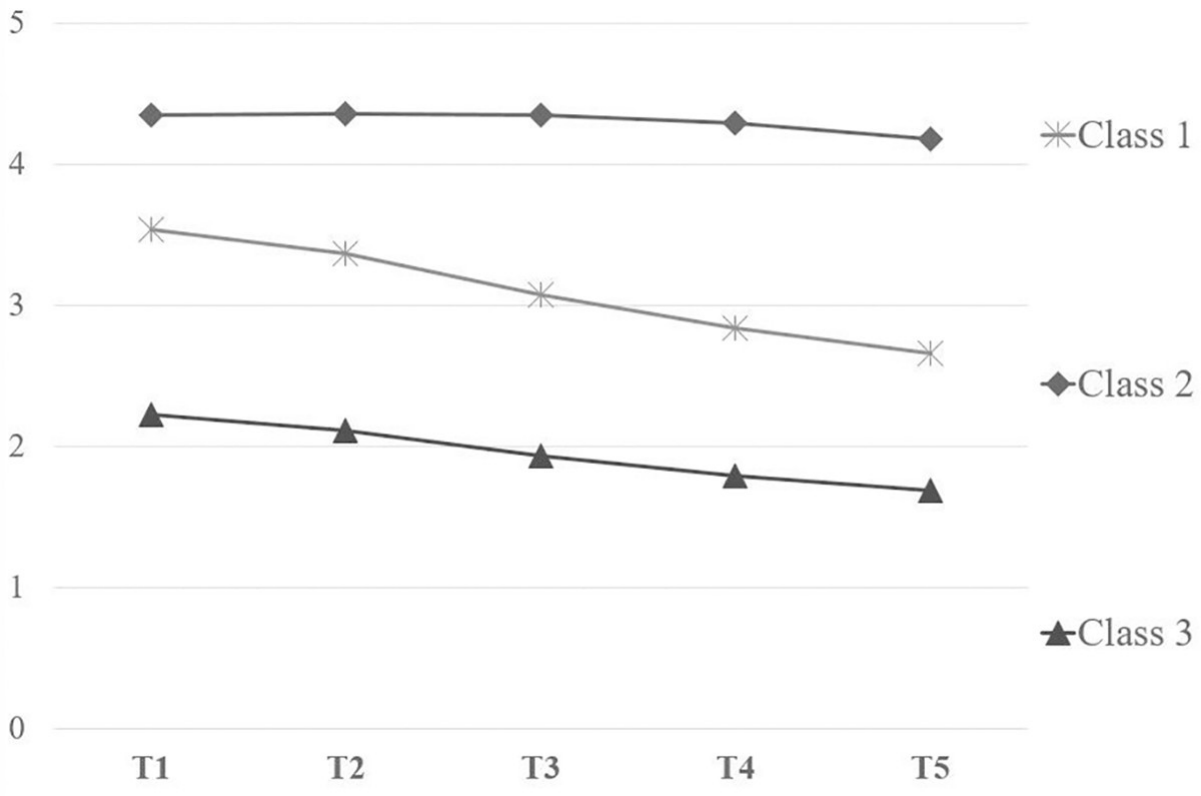
The three identified longitudinal trajectories of sexual dysfunction.

LCGA was performed on the sexual dysfunction scores over time. A three-class model with three distinct longitudinal trajectories was chosen because the LMR-LRT indicated that the three classes are significantly better than two (p < .001) and better than four (p = .404). Moreover, this three-class was also supported by a high entropy value (.913) and total count within each class above 1% (min = 15.49%; max = 59.92%). Fig 2 reports these identified longitudinal classes of change of sexual dysfunction over time.

Patients in the first class (24.59% of the total count) showed a medium initial level of sexual dysfunctionality (intercept = 3.54, p < .001) and a moderate recovery over time (slope = - 0.18, p < .001; quadratic = 0.01, p = .172). We labeled this class as “Class 1”. As shown in Table 3, 55.1% of patients presented with biopsy Gleason score 6, while in 44.9% of cases was between7 and 8. The majority of patients (71.2%) had a pre-surgical PSA between 4 and 10, and 77.6% had an ASA score between 2 and 3. The Charlson Index was lower than 1 in 72.9% of the sample. Moreover, 91.5% of patients who underwent RARP and belonging to this Class had not post-surgical complications. “Class 2” encompassed 59.92% of patients with the higher levels of sexual impairment at baseline (intercept = 4.34, p < .001) and a flat and non-significant recovery over time (slope = 0.02, p = .123; quadratic = - 0.01, p = .002). Almost eighty-two percent of patients belonging to Class 2 had a Gleason Score lower than eight, 63.2% of them had a PSA index varying between 4 and 10, and 84.2% an ASA score of 2 or 3. Their Charlson Index was lower than 1 in 69.4% of the cases and 87.5% of the Class 2 had not complications after surgery (see Table 3). The third class (15.49% of the total count), labeled “Class 3”, was characterized by the lowest levels of sexual dysfunction at 45 days after the RARP (intercept = 2.23, p < .001) and a moderate recovery over time (slope = - 0.11, p = .046; quadratic = 0.01, p = .420). Patients belonging to Class 3 showed a biopsy Gleason score of 6 in 66.2%, 7 in 26.8% and 8 in 7% of the cases, respectively. Seventy-five% of patients had a PSA index from 4 to 10 and an ASA score of 2 or 3. Eighty-six percent of patients had a Charlson index lower than 1, while almost 93% percent had not complications after RARP (See Table 3).

**Table 3:** Clinical variables in identified sexual clusters.

Clinical Variables	Class 1 (%)	Class 2 (%)	Class 3 (%)
**Gleason Score**			
≤ 6	55.1	37.6	66.2
7	35.6	44.3	26.8
8	9.3	12.2	7.0
9–10	0	5.9	0
**PSA Class**			
Less than 4	14.4	13.2	8.3
4–10	71.2	63.2	75.0
More than 10	14.4	23.6	16.7
**ASA Class**			
1	22.4	15.8	24.6
2–3	77.6	84.2	75.4
**Charlson Index**			
<1	72.9	69.4	86.1
≥1	27.1	30.6	13.9
**Complications**			
No	91.5	87.5	93.1
Yes	8.5	12.5	6.9

### Clinical predictors of longitudinal trajectories of sexual dysfunction

Then, the predictive role of clinical and sociodemographic variables to explain membership in longitudinal trajectories of sexual dysfunction was evaluated through multinomial logistic regression. Specifically, pre-surgery sexual dysfunction score, age, and nerve sparing were introduced in this model as predictors of longitudinal change membership.

Because “Class 2” was the worst longitudinal trajectory of sexual dysfunction over time, this class was chosen as the reference category in this multinomial regression model. Results showed that more elderly patients (B = - 0.08, OR = 0.93, p = .004) and higher level of pre-surgery sexual symptoms (B = - 1.30, OR = 0.27, p < .001) had a lower chance of belonging to the “Class 3” compared to the “Class 2”. Nerve sparing procedure was not able to distinguish between patients belonging to “Class 2” and “Class 3”. Again, compared to the “Class 2”, the “Class 1” was characterized by patients with lower levels of pre-surgery sexual problems (B = - 0.89, OR = 0.41, p < .001) and subjected to bilateral nerve sparing RARP (B = 1.19, OR = 3.29, p = .028). Age and unilateral nerve sparing RARP did not distinguish between patients belonging to “Class 2” and “Class 1”.

### Associations between membership in longitudinal trajectories of urinary incontinence and sexual dysfunction

The results of the Chi-square test assessing the association between memberships in urinary incontinence and sexual dysfunction membership demonstrated that the two cluster membership tended to co-occur [*X*^2^(8, N = 478) = 60.20, p < .001]. Specifically, the adjusted residual reported in the contingency table (Table 4) demonstrated that patients belonging to the “Class 1” of sexual dysfunction had a low chance to be ascribed to the “Class 4” and “Class 5” and a high chance to be ascribed to the “Class 1” of urinary incontinence. We highlighted a symmetrical and opposite pattern of associations for the “Class 2” of sexual dysfunction; specifically, patients in this longitudinal trajectory were more likely to be ascribed to “Class 4” and “Class 5” while they had a low likelihood to belong to the “Class 1”. Finally, patients in “Class 3” of sexual symptoms had a low likelihood to belong to “Class 3” and “Class 4” while showing a high probability to be ascribed in “Class 1”.

**Table 4:** Contingency table between longitudinal trajectories’ membership of urinary incontinence and sexual dysfunction (adjusted residuals within each cell are reported in Italics).

	Urinary incontinence					
		Class 1	Class 2	Class 3	Class 4	Class5
Sexual Dysfunction	Class 1	56	34	21	6	1
		*2*.*2*	*0*.*2*	*0*.*4*	*- 2*.*8*	*- 2*.*1*
	Class 2	81	82	54	52	19
		*- 5*.*8*	*0*.*3*	*1*.*5*	*4*.*7*	*3*.*2*
	Class 3	48	18	5	1	0
		*5*.*3*	*- 0*.*6*	*- 2*.*4*	- 3.1	*- 1*.*9*

## Discussion

This study identifies different longitudinal trajectories of patients with PC undergone RARP based on their initial status and change over time in urinary incontinence and sexual dysfunction.

Different trends for each of these two EPIC-26 subscales were identified: five and three classes were found for urinary incontinence and sexual dysfunction subscales, respectively. All but one of the trajectories of urinary incontinence showed a moderate recovery over one year after surgery. Only the class with the worst initial status showed a non-significant recovery over time: people who have a high level of urinary incontinence 45 days after surgery are likely to have a worse recovery. In fact, the small proportion of patients with high urinary incontinence rates at baseline either do not recover, or their symptomatology gradually decreases over time. On the contrary, patients with low level of leaking urine after RARP have a faster, and sometimes sudden, recovery. According to the literature [6], these different trends may be affected by sociodemographic and clinical variables, like age, pre-surgical condition and BMI. Elderly and overweight patients may display higher level of incontinence 45 days after surgery and may have more problems in the recovery trajectories, while those with lower levels of pre-surgery continence seem to have greater chance to recover faster and report very small problems one year after RARP.

Similar to urinary incontinence, sexual dysfunction presents different classes of post-surgery condition and recovery. Patients with low and moderate problems 45 days after surgery can face a moderate linear recovery, while men with more significant impotence immediately after surgery may take a longer period to solve sexual dysfunctions. In fact, most of patients display high level of impotence after RARP and with no recovery of their potency even after one year from surgery. Age and the pre-surgical sexual condition are important aspects to identify patients with difficulties in recovery from erectile dysfunction. In fact, elderly men and patients with sexual impairment before surgery are less likely to recover than the others. A recent study with a sample stratified by men’s pre-operative scores of erectile function, showed that each group statistically and differently improved in potency rates at consecutive follow-up visits up to 24 months, proving that the time of recovery varies along with patients’ baseline characteristics [31]. Moreover, bilateral nerve-sparing surgical intervention seems to positively reduce post-surgery recovery.

The evaluation of the association between membership in the longitudinal trajectories of urinary incontinence and sexual dysfunction may help physicians in the identification of patients with difficulties in the recovery of both symptoms. In fact, some of these patients reported both steady problems in sexual function and constant high levels of urinary incontinence over time. On the contrary, people with low rates in urinary incontinence, more probably will also display less level of sexual potency. Sexual and urinary dysfunctions are the most common consequences after PC treatments [8] and the displayed association underlines the importance of investigating these aspects in clinical practice. In fact, the scientific literature shows that sexual life and urinary incontinence are strictly related and the most bothersome aspects of incontinence were its effects on partner relationship and sexual life [12,15,41].

These results identify urinary incontinence and sexual dysfunction as the most common and unsolved drawbacks after one year from RARP. Even if they show an overall improvement over time, one year after RARP no patients’ class has a full recovery and most of the time scores down only a point in one year. For this reason, it would be important to analyze cancer survivors’ recovery for a longer time in order to better describe the complete process of care [42,43]. Research studies on short and long side-effects pointed out that patients still suffer from erectile dysfunction, but have a good continence status, even after a median follow-up of 42 months after surgery [12]. Moreover, physical impairments negatively impact patients’ level of distress, quality of life and life satisfaction even 2- or 3-years after diagnosis [27,44].

### Clinical implications

The identification of different longitudinal trajectories of patients with PC undergone RARP in urinary incontinence and sexual dysfunction provides new evidences on patients’ recovery over the care process. These evidences may be important elements to be discussed during patient-physician relationship: urologists may adopt this information to help men make informed decisions in line with their individual preference and adjust their expectations about long-term sexual life. In fact, physicians’ and patients’ hopes of body function recovery do not always concur and great clarity would be needed [45,46].

Thanks to the adoption of a person-oriented approach, our results may be useful to identify patients at risk and typical trajectories of recovery, which are important prerequisites of a patient-centered care and planned healthcare programs. A patient-centered approach related to a multidisciplinary cooperation would be important to overcome medical barriers and empower patients, making them aware of their care pathway [47,48]. Along with personalized interventions and the development of eHealth platforms to enhance patient’s health literacy and engagement [49–51], a new approach to the healthcare system would be needed. The implementation of a new healthcare system based on value would help set up predictive and individualized care pathways for each cancer diagnosis. The patient would be followed along the care process collecting psychological, medical outcomes and economical outcomes in order to implement predictive model of care [52–54].

### Limitations

Several limitations of this study have to be considered. First of all, we were not able to conduct a growth mixture modelling (GMM) analysis instead of the LCGA, although a larger sample would have been necessary to conduct a GMM. In LCGA we set to 0 the intra-class variances of intercepts and slopes, providing a less accurate estimate of the latent trend of the dysfunctions’ recovery [55]. Moreover, comorbidities and other possible psychological or medical predictors have not been collected. Finally, the measured outcomes were collected up to only one year after surgery: it would be important to extend the follow-ups, in order to better analyse patient’s recovery of functions, which mostly lasts more than one year after treatments [6,27].

Therefore, our future directions would be to collect more information about patient’s characteristics and psychological outcomes through the use of standardized questionnaires and semi-structured interviews to provide more comprehensive framework of the patient care process.

## Conclusions

Patients who underwent RARP and followed for one year after surgery showed different trajectories of recovery both in urinary incontinence and in sexual dysfunction. The LCGA identified three and five different initial medical status and trends of recovery in impotence and urine leaking, respectively, that were influenced by clinical and sociodemographic predictors such as age, pre-surgical scores of physical dysfunctions, diabetes and BMI. This study highlights different categories of patients at risk who may be important to know in order to develop personalized medical pathways and predictive models in a value-based healthcare.

## Supporting information

S1 Dataset (SAV)

## Abbreviations

BMI: Body Mass Index
EPIC-26: Expanded Prostate Cancer Index Composite (Short Form)
LCGA: Latent Class Growth Analysis
LMR-LRT: Lo-Mendell-Rubin likelihood ratio test
PC: Prostate Cancer
RARP: Robot-Assisted Radical Prostatectomy
